# The Reason Why rTMS and tDCS Are Efficient in Treatments of Depression

**DOI:** 10.3389/fpsyg.2019.02923

**Published:** 2020-01-13

**Authors:** Milena Čukić

**Affiliations:** ^1^Department for General Physiology and Biophysics, University of Belgrade, Belgrade, Serbia; ^2^Instituto de Tecnología del Conocimiento, Complutense University of Madrid, Madrid, Spain

**Keywords:** physiological complexity, rTMS, tDCS, depression, efficiency of treatment, neuromodulation

## Introduction

The exact neurophysiological mechanisms of repetitive transcranial magnetic stimulation (rTMS) and transcranial direct current stimulation (tDCS) for treating patients diagnosed with depression are still not clear. Results of previous structural and functional MRI studies showed an aberated functional connectivity in major depressive disorder (MDD) (Vederine et al., 2011; de Kwaasteniet et al., 2013). Those, as well as several connectivity studies (Bluhm et al., 2009; Berman et al., 2011; Zhang et al., 2011; Kim et al., 2013; Chen et al., 2015) seem to support the hypothesis that aberrant functional connectivity within fronto-limbic system underlies the pathophysiology of depression. It should be noted that antidepressant application of both rTMS and tDCS is based on previous findings that these two methods help in the case of hypoactivity of the left dorsolateral prefrontal cortex (DLPFC) (Grimm et al., 2006). Those structural and functional differences probably introduce abnormal physiological complexity demonstrated in electroencephalographic (EEG) (Ahmadlou et al., 2012; Bachmann et al., 2013; Hosseinifard et al., 2014; De la Torre-Luque and Bornas, 2017; Jaworska et al., 2018; Lebiecka et al., 2018) as well as in electrocardiographic (ECG) signals in depression (Migliorinni et al., 2012; Rossi et al., 2016; Iseger et al., 2019).

TDCS is low-intensity modality of transcranial electrical stimulation (TES) which induces very mild sensations in the skin (Stagg and Nitsche, 2011). Much later developed TMS primarily uses a strong magnetic field to induce an electric field in the cortex painlessly, initiating optimally focused activation of neural structures (Barker et al., 1985). Some of its modalities used in psychiatry are repetitive TMS (rTMS) and intermittent theta burst TMS (iTBS). In the present abundant literature about both rTMS and tDCS, there is scarce evidence of *why* these two techniques are capable of ameliorating depressive symptoms. We still don't know what precise mechanisms behind them are. Only a fraction of published research (Amassian et al., 1989; Maccabee et al., 1990; Wassermann and Grafman, 2005; Miranda et al., 2009; Ilmoniemi and Kičić, 2010; Alam et al., 2016) describe the theoretical background of those mechanisms from electromagnetics/physics point of view. The majority of published studies are based on multi-centric comparisons of clinical efficiency (Brunoni et al., 2016; Antal et al., 2017; Mutz et al., 2018) and computational methods-or simulations (Miranda et al., 2001, 2006; Wagner et al., 2007; Huang et al., 2017). Recently, a team of leading researchers in low intensity electrical transcranial stimulation reviewed clinical outcomes for 8,000 people (Antal et al., 2017) confirming its safety and effectiveness, and defined the regulatory and application guidelines for future research.

A term “non-invasive” (attached to both rTMS and tDCS) stems from obsolete medical point of view that the stimulating electrodes do not enter the crania (and the stimulation is performed either via small electrical charges in case of tDCS or via Faraday's induction). The real effect of “non-invasive” electromagnetic stimulation (rTMS and tDCS) cannot be measured directly due to their non-invasive nature. Opitz stated in recent research, that the important point is in interpretability of stimulation effects (Opitz et al., 2015): “if electric fields are delivered inconsistently, but effects are observed nevertheless, the results are more difficult to interpret because effect could be driven by other incidentally affected brain regions.” Both tDCS and TMS are shown to initiate these “unintended” effects: Bestmann showed using MRI that TMS of motor cortex below the threshold power can activate some other deeper structures, contrary to previous belief and Li showed similar phenomena in the case of tDCS (Bestmann et al., 2003, 2004; Li et al., 2018).

The hypothesis here is that both non-invasive electromagnetic modalities of brain stimulation, rTMS and tDCS, are efficient in depression treatments because of *their proven ability to decrease the physiological complexity* (Čukić et al., 2013, 2019a; Lebiecka et al., 2018; Zuchowicz et al., 2019). The hallmark of MDD is elevated physiological complexity of EEG measured by various entropy measures, fractal dimension, symbolic dynamic approach measures, geometric techniques like recurrence plots and other measures stemming from complex systems dynamics theory (De la Torre-Luque and Bornas, 2017). There are also findings that link changes in heart rhythm complexity with depression (Migliorinni et al., 2012) and the outcomes of rTMS treatment(Royster et al., 2012; Lebiecka et al., 2018; Iseger et al., 2019).

**Figure 1:**
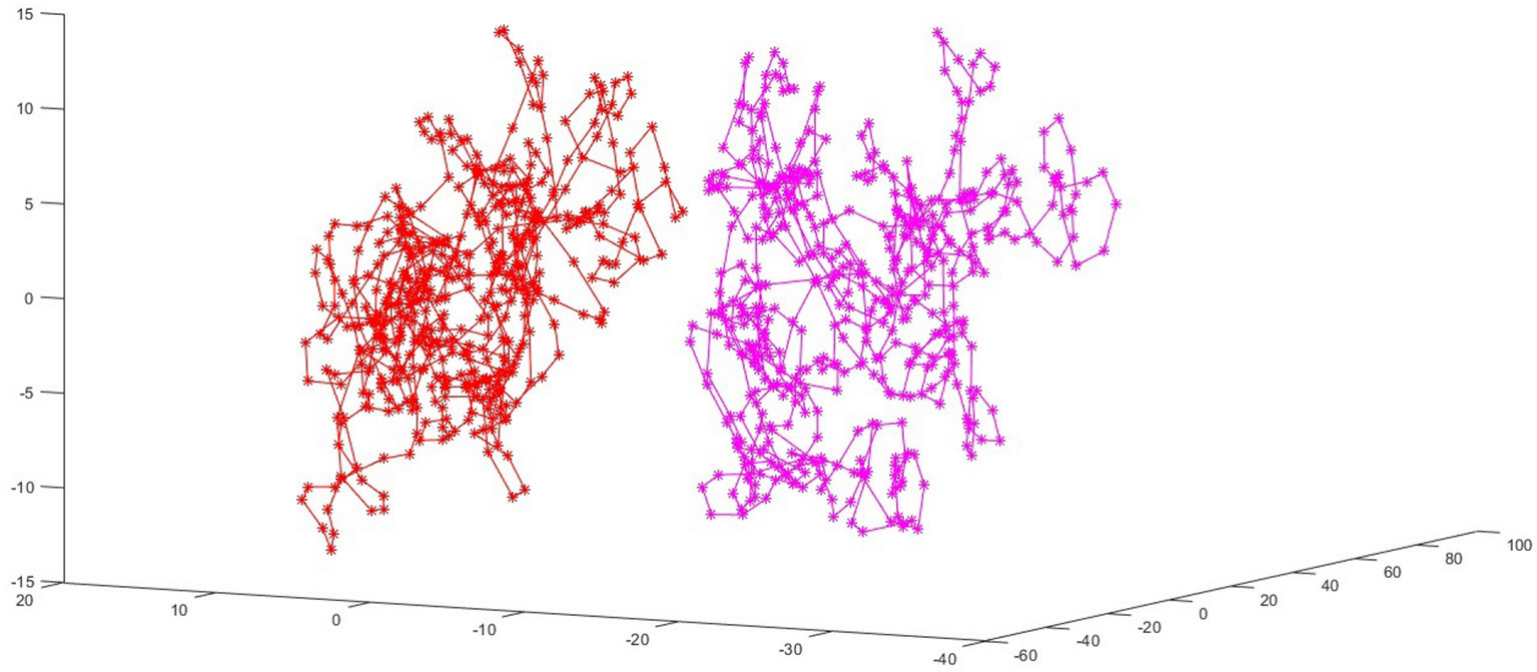
The red voltage samples are taken from the EEG recording before the stimulation, and pink ones from 32 min after the tDCS stimulation. The first three principal components of raw EEG samples before and after tDCS stimulation are illustrating that they belong to separate parts of the phase space. Here is a PCA plot for person number 8, with cathodal (C) stimulation. This figure is part of results published in Chapter 3 in book (Čukić Radenković, 2019), but this particular PCA plot is not displayed before (due to limited space in previous publication).

The evidence supporting the close relationship between the electrophysiological complexity, depressive symptoms, and rTMS and tDCS treatment is sufficient but veiled. First, in our 2011 study we showed that even a single pulse transcranial magnetic stimulation (spTMS) can decrease the complexity of electrophysiological signal (Čukić et al., 2012, 2013). Second, Mutanen et al. (2013) used Global Recurrence analysis on concurrently recorded EEG to show that TMS is capable of inducing a “brain-shift” after the stimulation., that is moving the system of brain networks to higher-energy less-probable state in healthy controls. Based on this work we applied the same method but with tDCS (Čukić et al., 2018b, 2019a,b). Čukić et al. (2018b) showed for the first time the graphical representations of tDCS-induced “brain-shift” obtained by principal component analysis (PCA) applied on raw EEG signal samples. PCA was used in our data mining projects to check for separability of data for later classification. This study re-used EEG signals from 16 healthy controls recorded during cathodal and anodal tDCS stimulation protocols from Pelliciari et al. (2013) (which is also elaborating on the difference between cathodal and anodal stimulation). Obtained PCA plots are showing that more than a half an hour post stimulation the system is still in higher-energy lower-probable state “brain-shift” due to the tDCS stimulation. The first three principal components of raw EEG samples before and after tDCS stimulation are illustrating that they belong to separate parts of the phase space. One of participants PCA plot after cathodal stimulation is shown in Figure 1.

Several researchers who used various non-linear measures of complexity of EEG confirmed that physiological complexity is elevated in MDD (Ahmadlou et al., 2012; Bachmann et al., 2013, 2015, 2018; Faust et al., 2014; Hosseinifard et al., 2014; Akar et al., 2015; Čukić et al., 2018a, 2019a; Lebiecka et al., 2018). One of the most inclusive review studies on various spectral, fractal and other non-linear measures of relationship between physiological complexity and MDD, concluded that EEG signals in MDD are “probably more random than more complex” compared to those of healthy persons (De la Torre-Luque and Bornas, 2017). This might be due to impaired intrinsic feedback mechanisms important for many regulatory functions (Goldberger et al., 2002). This kind of abnormal functional connectivity is reported in several research papers from seemingly unrelated disciplines, like graph theory application in EEG connectomics (Lee et al., 2011; Van Essen et al., 2012; Castellanos et al., 2013; Kim et al., 2013), and Granger causality applied on fMRI signals (Hamilton et al., 2011). The fMRI and Fractional anisotropy (FA) research also found that within fronto-lymbic system there is abnormal functional connectivity in MDD (Vederine et al., 2011; de Kwaasteniet et al., 2013). De Kwaasteniet found that uncinate fasciculus, important for connecting prefrontal with limbic system, is not fully functional in MDD patients (de Kwaasteniet et al., 2013). Moreover, several studies examining connectivity in MDD found a different dynamical features, and several different regions (anterior cingulate cortex, insula, cingulate and hippocampal network) were confirmed as candidates for these differences (Mayberg, 1997; Mayberg et al., 1997, 1999; Bluhm et al., 2009; Berman et al., 2011; Ge et al., 2019). It is challenging to compare these findings since their methodological approaches are different in so many aspects. Also, Mendez et al. (2012) detected a higher focus on local connections than on global ones in MDD. This can also be seen in persons with depression in remission: previously detected abnormal functional connectivity decreases (Mendez et al., 2012). Lebiecka et al. (2018) showed that elevated physiological complexities diminished after treatment in those MDD patients that reacted well on rTMS (as measured by the decrease in complexity corresponding to remission scores after the treatment was measured) (see also Jaworska et al., 2018). Iseger et al. (2019) also revealed the connection between successful iTBS applied to the DLPFC and modulation of autonomic nervous system (Iseger et al., 2019).

Bestmann et al. (2004) demonstrated that with TMS application below the motor threshold power, MRI can detect a response from areas that were not intended to be stimulated (Bestmann et al., 2004). Li et al. (2018) were the first research group to demonstrate that tDCS can activate some structures within DMN. Opitz et al. (2015) conclude in their work that even the conductivity constants (dielectric constants for tissue types) used for calculating the effect of stimulation, or simulation, are not adequate for describing the much more demanding reality. Opitz's team detected both higher and lower actual values measured directly (with the array of implanted electrodes in patients that were candidates for surgical intervention on epileptic foci) than those predicted with standard simulation procedures for TES (Opitz et al., 2015, 2018). The effect of a stimulation can depend on the geometrical shape of the surface of sulci, which cannot be monitored during the use of a non-invasive procedure, and that also can lead to major miss-predictions (Čukić, 2006; Čukić et al., 2009; Saturnino et al., 2015; Alekseichuk et al., 2018; Opitz et al., 2018).

Although it can seem impossible to compare the two non-invasive brain stimulation techniques that are so different in the sense of their electromagnetic properties and the level of power they can induce in the living tissue, we can still recognize the same functional pattern. In many review papers exploring the efficiency of both rTMS and tDCS in clinical applications (Brunoni et al., 2016; Antal et al., 2017; Mutz et al., 2018), the conclusions are in line: they are effective, and tDCS can be applied even in primary care, but also as a maintenance treatment for already successful rTMS (Mutz et al., 2018). In a study examining the effect of electroconvulsive therapy, it is demonstrated that multiscale entropy is changed after the treatment (Okazaki et al., 2013), pointing again at the link between complexity changes and the effective treatment for depression. Zuchowicz et al. (2019) reported on detected synchronization of EEG as a feature of successful rTMS which is pointing at reduction of complexity, too.

For all electromagnetic stimulation treatments, the effect is of temporary nature. The rationale is that they can at least ameliorate the symptoms for a limited time; after which they need to be repeated. The common advantage of non-invasive brain stimulation techniques over medications is that there are no foreseeable harmful side-effects (Antal et al., 2017).

Although study of physiological complexity changes is still in the realm of research and mainly not in use in clinical setting, it is expected that soon clinicians would start using varying electromagnetic modalities of stimulation with better understanding of how they work—as means to decrease complexity characteristic of depression. Further research based on empirical data is necessary before making the final conclusion that non-invasive brain stimulation treatments may work through changing physiological complexity.

## Conclusion

To conclude, after all above mentioned results of various lines of research that tried to bring us closer to understanding various aberrations of depression, both rTMS and tDCS might be efficient because of their ability to decrease characteristically elevated levels of physiological complexity in depression.

## Author Contributions

MČ conceived the idea about the article, performed a literature research and wrote entire text.

### Conflict of Interest

The author declares that the research was conducted in the absence of any commercial or financial relationships that could be construed as a potential conflict of interest.
